# A Novel High-Throughput Screening Method for a Human Multicentric Osteosarcoma-Specific Antibody and Biomarker Using a Phage Display-Derived Monoclonal Antibody

**DOI:** 10.3390/cancers14235829

**Published:** 2022-11-26

**Authors:** Takuma Hayashi, Naoki Yamamoto, Gene Kurosawa, Kaori Tajima, Mariko Kondo, Noriko Hiramatsu, Yu Kato, Miho Tanaka, Hisateru Yamaguchi, Yoshikazu Kurosawa, Harumoto Yamada, Nobuyuki Fujita

**Affiliations:** 1 Department of Orthopedic Surgery, Fujita Health University, Toyoake 470-1192, Japan; 2 Support Office for Bioresource Research, Research Promotion Headquarters, Fujita Health University, Toyoake 470-1192, Japan; 3 International Center for Cell and Gene Therapy, Research Promotion and Support Headquarters, Fujita Health University, Toyoake 470-1192, Japan; 4 Perseus Proteomics Inc., Tokyo 153-0041, Japan; 5 Center for Joint Research Facilities Support, Research Promotion and Support Headquarters, Fujita Health University, Toyoake 470-1192, Japan; 6 Yokkaichi Nursing and Medical Care University, Yokkaichi 512-8045, Japan; 7 Institute for Comprehensive Medical Science, Fujita Health University, Toyoake 470-1192, Japan

**Keywords:** phage library, multicentric osteosarcoma, cell surface antigen, CAVIN1/Polymerase I and transcript release factor (CAVIN1/PTRF)

## Abstract

**Simple Summary:**

Human multicentric osteosarcoma (HMOS) is a variant of human osteosarcoma (HOS) in which multiple bone lesions occur simultaneously or asynchronously before lung metastasis. HOS is associated with a poor prognosis. Using the human antibody phage library, we obtained 95 types of antibody clones against HMOS cell lines and developed a fluorescence probe-based enzyme-linked immunosorbent assay (ELISA) that enables high-throughput screening of the reactivity of these antibodies. The lysate of the HMOS cell line was incubated with the selected antibody, and the antigen–antibody complex was recovered using magnetic beads. Liquid chromatography-mass spectrometry (LC/MS) analysis of the sample bands extracted from gel electrophoresis detected CAVIN1/PTRF specifically in HMOS cells. This novel high-throughput screening assay using antibody–drug conjugate targeting for these specific proteins is promising for clinical applications.

**Abstract:**

Osteosarcoma is a malignant tumor that produces neoplastic bone or osteoid osteoma. In human multicentric osteosarcoma (HMOS), a unique variant of human osteosarcoma (HOS), multiple bone lesions occur simultaneously or asynchronously before lung metastasis. HMOS is associated with an extremely poor prognosis, and effective treatment options are lacking. Using the proteins in our previously generated HMOS cell lines as antigens, we generated antibodies using a human antibody phage library. We obtained antibody clones recognizing 95 independent antigens and developed a fluorescence probe-based enzyme-linked immunosorbent assay (ELISA) technique capable of evaluating the reactivity of these antibodies by fluorescence intensity, allowing simple, rapid, and high-throughput selection of antibody clones. These results were highly correlated with those using flow cytometry. Subsequently, the HMOS cell lysate was incubated with the antibody, the antigen–antibody complex was recovered with magnetic beads, and the protein bands from electrophoresis were analyzed using liquid chromatography-mass spectrometry (LC/MS). CAVIN1/polymerase I transcript release factor was specifically detected in the HMOS cells. In conclusion, we found via a novel high-throughput screening method that CAVIN1/PTRF is an HMOS-specific cell membrane biomarker and an antigen capable of producing human antibodies. In the future, antibody–drug conjugate targeting of these specific proteins may be promising for clinical applications.

## 1. Introduction

Human osteosarcoma (HOS) is a malignant tumor that produces neoplastic bone or osteoid, with a high prevalence among teenagers. It is considered the most common malignant primary bone tumor [1]. There has been an increase in the number of patients with cancer as the population ages. Mortality directly associated with bone metastasis of a malignant tumor is rare; however, bone metastasis severely compromises the patient’s quality of life through pain, hypercalcemia, and limited mobility [2]. The prognosis remains poor in cases with ineffective chemotherapy, and lung metastasis is the primary cause of mortality. Lung metastasis has been observed in approximately 25% of HOS [1]. The five-year survival rate for HOS has reached 60–70% owing to recent advances in chemotherapy, diagnostic imaging, and surgical therapy [3,4]. However, from a clinical perspective, prevention of bone metastasis was associated with further improvements in survival and quality of life.

Metastasis of malignant tumors is known to have tissue directivity. Breast, prostate, and lung cancers are examples of tumors that readily metastasize. The “seed and soil” hypothesis has been put forward, which states that organ-selective metastasis of a tumor requires a mutual relationship between the biological environment of the tumor cells and the target organ [5]. Metastasis of a malignant tumor undergoes several steps: proliferation of tumor cells in the primary lesion, vascular infiltration, dissociation from the primary site, binding to the target organ, and proliferation at the new site [6]. Many researchers have investigated the factors that govern the affinity of tumors to bone. They reported the involvement of various factors in bone metastasis, including bone morphogenetic protein, interleukin, and cadherin [2,7,8,9,10,11].

Two types of HOS metastasis have been reported: HOS without bone metastasis and osteosarcoma with bone metastasis. HOS with bone metastasis showed increased expression of CD31, which is involved in migration between endothelial cells, and bone morphogenetic protein (BMP), which induces and activates various mesenchymal cells that affect bone formation. On the other hand, the expression of the Met/hepatocyte growth factor (Met/HGF) receptor, which promotes HOS differentiation, was increased in HOS without bone metastasis [12]. HOS cells have been reported to express αvβ3 integrin as a ligand for CD31 [13,14]. The homophilic interaction between CD31–CD31 and the heterophilic interaction between CD31–αvβ3 integrin suggested that CD31 expression may promote HOS cell adhesion to and migration between endothelial cells [12]. BMPs belong to the transforming growth factor-β (TGF-β) superfamily of proteins. BMPs, named after their ability to induce ectopic bone formation, have been implicated in BMP signaling in the development and progression of musculoskeletal carcinoma [15]. BMP ligands and receptors are expressed in most HOS cell lines, but BMP signaling activity is not decreased in HOS compared to benign bone-forming tumors, suggesting that BMP signaling activity is not a major factor in the pathogenesis of HOS. BMP signaling in HOS may be associated with a worse prognosis. At the cellular level, BMP signaling appears to mediate migratory effects in HOS and chondrosarcoma cell types, possibly via integrin β1 interaction and activation [16]. Vascular endothelial growth factor (VEGF) expression and high vascular distribution within HOS may correlate with a poor prognosis, suggesting a higher rate of cancer recurrence and a higher frequency of distant metastases in high VEGF groups [17].

In a particular variant of HOS, multiple bone lesions occur simultaneously or asynchronously before lung metastasis. This variant is referred to as human multicentric osteosarcoma (HMOS), which has been reported to have an incidence of 1.5–3.6% among all HOS cases [18,19,20,21]. While the pathological characteristics of HMOS remain mostly unclear, the common notion is that it is a specific form of multiple bone metastasis from tumor cells with a strong affinity to the bone. We previously reported a case of HMOS in which we harvested tissues from a lesion site on the left humerus, assumed to be the primary lesion, and a lesion on the sternum that had occurred later during chemotherapy. We established HMOS cell lines from the two lesions [22]. We then compared the two cell lines and observed a higher expression of the chemokine CXCR4, which is believed to be involved in HOS metastasis, in the secondary (HMOS-P) as compared to the primary lesion cell line (HMOS-A). This suggests that the secondary lesion likely metastasized from the primary lesion [22].

The human antibody library termed “Antibody Intended for Multiple Screening” (AIMS) and the related methodology suite have been well established by the Division of Antibody Project of the Fujita Health University, and have been used in several studies to develop cancer therapeutic antibodies [23,24,25,26,27]. The AIMS human antibody library comprises approximately 1 × 10^11^ clone types obtained from human B-lymphocytes using the phage display method. With this library, an antibody against a specific target antigen or an unknown antigen may be isolated. The antibodies are fully human and are expected to have clinical applications as anti-tumor drugs [24]. However, when obtaining an antibody using the phage display system, there may be several hundreds of antibody types for one type of cell. A lot of time and effort is required to investigate each antibody individually.

In this study, we performed a comprehensive antibody screening of HMOS-P surface antigens using a phage display method. We established an antibody selection method using a fluorescence probe-based enzyme-linked immunosorbent assay (ELISA), which has a higher throughput than the conventional analytical method of flow cytometry (FCM). We further aimed to identify, isolate, and characterize those antibodies strongly expressed with bone affinity in HMOS cell lines but weakly expressed in HOS cell lines. We thus attempted to identify potential novel biomarkers for HMOS diagnosis.

## 2. Materials and Methods

### 2.1. Cell Lines

We established two new HMOS cell lines (Supplementary Figure S1), one of which was derived from the anterior (i.e., primary) lesion after an incisional biopsy of the left humerus (designated as HMOS-A), and the other was derived from one of the posterior (i.e., secondary) lesions of the sternum (designated as HMOS-P) [22]. To compare the cell lines, we used the following HOS cell lines: MNNG-HOS (EC87070201; European Collection of Authenticated Cell Cultures: ECACC, Salisbury, UK), MG63 (EC86051601; ECACC), and Saos2 (EC89050205; ECACC). MNNG-HOS, MG63, and Saos2 cells were cultured in accordance with the culture conditions of ECACC. HOS is defined as malignant tumors that form osteoids. Per this definition, NOS-1 (RCB1032) and NOS-2 (RCB1033) HOS cell lines that form osteoids in vitro or in xenograft models were used to validate HMOS-specific proteins. NOS-1 and NOS-2 cells were provided by the Riken BioResource Research Center (RIKEN BRC) through the National BioResource Project of the Ministry of Education, Culture, Sports, Science and Technology (MEXT), Japan. Mycoplasma contamination in the cell lines was inspected using a mycoplasma detection kit (EZ-PCR^TM^ Mycoplasma Test Kit, Biological Industries, Cromwell, CT, USA), according to the manufacturer’s instructions. This study was approved by the Ethics Committee of Fujita Health University (approval number: 15-181).

### 2.2. Antibody Library and Screening

The human antibody library (AIMS7 phage library) and the screening method of the Division of Antibody Project of Fujita Health University Institute for Comprehensive Medical Science were used [23,27]. Briefly, cultured HMOS-P cells were washed and incubated with 1 × 10^13^ colony-forming units (CFU) of AIMS human antibody library phage at 4 °C for 4 h with constant rotation. The final concentration of the reaction liquid was then divided into two parts, each of which was layered onto 0.6-mL organic solvent (dibutyl phthalate:cyclohexane = 9:1) and centrifuged. The precipitated cells and phage complex were snap-frozen in liquid nitrogen and thawed at 37 °C to yield the phage.

The obtained phage was added to *E. coli* DH12 cultured in yeast extract-tryptone culture medium at 37 °C for 1 h. The phage-infected *E. coli* was cultured for 12–15 h at 30 °C. A 5-mL aliquot of the culture solution was mixed with KO7 helper phage and cultured. This culture solution was then centrifuged at 8000 rpm (11,800× *g*) for 10 min, the supernatant was collected, mixed with polyethylene glycol solution (20% polyethylene glycol 6000, 2.5 M NaCl), and centrifuged to precipitate the phage. The phage was then suspended in phosphate-buffered saline (PBS) to provide the first screening phage. Overall, four screening phages were collected, and the set of antibodies obtained from the fourth screening was used for further experiments (Supplementary Table S1).

Ninety-five clones were selected for the fourth HMOS-P screening. All isolated clones encoding antibodies were sequenced and classified according to the data summarized in IgBLAST [28]. According to the classification of respective VH and VL nucleotide sequences, candidate monoclonal antibodies were selected for analysis.

### 2.3. Analysis of Fluorescence Probe-Based ELISA

HMOS-P cells (4 × 10^4^) were seeded onto a 96-well assay plate (Corning Inc., Corning, NY, USA) and cultured for 24 h. The primary antibody, i.e., the phage solution obtained from the antibody screening (antibody solution), was incubated with the cells for 1 h at 37 °C. The cells were washed with PBS and incubated with the secondary antibody, anti-mouse cp3 polyclonal antibody (1:370 dilution), for 30 min at 37 °C. The cells were then washed with PBS and incubated with the third antibody, Alexa 488 anti-mouse IgG (Molecular probes; 1:1000 dilution), for 30 min at 37 °C. The cells were washed twice with PBS, and the fluorescence intensity was measured using ARVO X (PerkinElmer Inc., Waltham, MA, USA). Fluorescence intensity was measured at 25 sites per well, and values ≥ 10,000 arbitrary units (AU) were considered positive. Using the “positive” antibodies, ELISA analyses were repeated in a similar fashion using HMOS-A, MNNG-HOS, MG63, and Saos2 cell lines. The analyses were performed five times in each case, and the fluorescence intensity was compared using the median value from the five replicates.

### 2.4. Flow Cytometry Analysis

The antibodies judged to be positive by ELISA were used as the primary antibodies. Cultured HMOS-A, HMOS-P, MNNG-HOS, MG63, and Saos2 cells were dissociated with TrypLE^TM^ Select (Thermo Fisher Scientific K.K., Waltham, MA, USA) and washed with PBS. The primary antibodies selected based on the ELISA screens (10, 12, 17, 19, 43, and 77) were diluted at 1:10 and incubated with the cells for 20 min at 4 °C. The cells were then washed with PBS. The cells were then incubated with the secondary anti-rabbit cp3 polyclonal antibody (1:370 dilution) and the third Alexa 488 anti-rabbit IgG (1:1000 dilution) for 20 min each at 4 °C. The cells were washed with PBS in the dark and analyzed using FACScan^TM^ (Becton Dickinson and Company, Franklin Lakes, NJ, USA). Each measurement of the individual primary antibodies was repeated thrice, and the positive rate with respect to the negative control and the GeoMean value of fluorescence intensity were calculated using CellQuest^TM^ Pro (Version 5.2.1, Becton Dickinson and Company, Waltham, MA, USA).

### 2.5. Immunohistochemistry

Paraffin sections of the HMOS posterior lesion site and HOS, from five patients, were immunohistochemically stained using hematoxylin and eosin (HE) and primary antibodies 10, 12, 17, 19, 43, and 77. Before staining, the antigen was retrieved by immersing the paraffin sections in a citric acid buffer with pH 6.0 and heated twice at 90 °C for 20 min in a microwave. The sections were incubated with a 1:10 dilution of each of the six “positive” primary antibodies for 1 h, washed with PBS, and incubated with the secondary anti-mouse cp3 polyclonal antibody (1: 370 dilution) at 37 °C for 30 min. The sections were washed with PBS and incubated with Histofine^®^ Simple Stain^TM^ MAX-PO MULTI (Nichirei Corporation, Tokyo, Japan) at 37 °C for 30 min. The sections were then stained with liquid 3,3′-diaminobenzidine tetrahydrochloride (DAB) + Substrate Chromogen System (Dako), and cell nuclei were stained with hematoxylin. Finally, the sections were observed under a BX51 upright fluorescence microscope fitted with a DP71 digital camera (Olympus Corporation, Tokyo, Japan).

### 2.6. Immunoprecipitation with Magnetic Beads and Electrophoresis

The HMOS-P cell line was lysed using the ULTRARIPA^®^ Kit (BioDynamics Laboratory Inc., Tokyo, Japan) to prepare cytoplasmic and cell membrane lysates. The lysate was individually incubated with antibody number 12, and an anti-rabbit cp3 polyclonal antibody was added as the secondary antibody. The antigen–antibody conjugates were adsorbed by incubating with µMACS™ Protein A/G MicroBeads (Miltenyi Biotec BV & Co. KG, Gladbach, Germany). This reaction solution was then incubated with a MultiMACS™ Protein A/G kit (Miltenyi Biotec BV & Co. KG), and the antigen–antibody complex was washed and concentrated by immunoprecipitation using magnetic beads. The antigen–antibody complex was then cleaved by enzymes, released from the magnetic beads, and eluted from the column.

The cell lysate, the negative control (with only the antibody), and the antigen–antibody complex incubated with the cell lysate were subjected to sodium dodecyl sulfate-polyacrylamide gel electrophoresis (SDS-PAGE). The gels were stained with SYPRO^®^ Ruby Protein Gel Stain (Thermo Fisher Scientific K.K.), and bands were detected using FLA3000G (FUJIFILM Healthcare Laboratory Co., Ltd., Tokyo, Japan). Finally, the Coomassie-stained gels were used to visualize the regions where the antigen–antibody complex was detected.

### 2.7. Protein Digestion and LC/MS Analysis

The gel was cut into smaller bands that reacted differently than the bands detected by electrophoresis of the negative control, i.e., with antibody only. After washing the gel fragments, they were de-stained with 50 mM ammonium bicarbonate (NH_4_HCO_3_) containing 50% acetonitrile (MeCN). The gel fragments were dehydrated with acetonitrile and dried in a vacuum evaporator. Cysteine residues were reduced using 10 mM DTT/50 mM NH_4_HCO_3_, followed by alkylation with 8 µL of 500 mM iodoacetamide (IAA) in 50 mM NH_4_HCO_3_. The gel fragments were washed and dehydrated again, and dried gel fragments were digested with 200 ng of trypsin (Promega Corporation, Madison, WI, USA) in 50 mM NH_4_HCO_3_ at 37 °C for 16 h. The trypsinized gel extract with 50% MeCN/0.1% trifluoroacetic acid (TFA) was pooled and desalted using GL-Tip SDB (GL Sciences Inc., Tokyo, Japan). The samples were processed using a solution eluted with 80% MeCN/0.1% TFA.

The samples were analyzed by liquid chromatography (LC, EASY-n LC 1000, Thermo Fisher Scientific) coupled to a mass spectrometer (MS, Orbitrap Fusion™ Tribrid™ Mass Spectrometer, Thermo Fisher Scientific). The AcclamPepMap100 trapping column (#164946, Thermo Fisher Scientific) and EASY-Spray LC columns (#ES800A, Thermo Fisher Scientific) were used. The 60-min gradient program was performed with water containing 0.1% formic acid (FA) and MeCN with 0.1% FA at a flow rate of 300 nL/min. Data analysis was performed using Proteome Discoverer™ version 2.2 (Thermo Fisher Scientific) with MASCOT (Matrix Science, Boston, MA, USA) as the search engine and SwissProt HomoSapience (v2017-10-25) as the database. The Human Protein Atlas (www.proteinatlas.org (accessed on 1 November 2022)) was a freely available interactive resource, and was used as a reference for the survey of candidate proteins resulting from the analysis [29].

### 2.8. Confirmation with Purified Protein and Commercial Antibody

Purified commercially available purified CAVIN1/PTRF (LifeSpan Biosciences, Inc., Seattle, WA, USA) was immobilized on a microplate and incubated with antibody number 12. After washing with PBS, an anti-mouse cp3 polyclonal antibody was added. After washing with PBS, an anti-mouse goat IgG-HRP (Santa Cruz Biotechnology, Inc., Santa Cruz, CA, USA) HRP-labeled secondary antibody was added. After washing with PBS, the absorbance at 450 nm was measured with a microplate reader (Benchmark, Bio-Rad Laboratories, Inc., Hercules, CA, USA) using a tetramethylbenzidine staining kit (Nacalai Tesque, Inc., Kyoto, Japan). Instead of the primary antibody, the reaction with PBS was measured as a blank.

As a confirmatory experiment, immunostaining of HMOS and HOS tissues (tissues from three patients) was performed using the commercial antibody against CAVIN1/PTRF (Proteintech Group, Inc., Rosemont, IL, USA), which was diluted at 1:100 and incubated at 37 °C for 30 min. The sections were washed with PBS and incubated with Histofine^®^ Simple Stain^TM^ MAX-PO MULTI at 37 °C for 30 min. The sections were stained with a liquid DAB+ Substrate Chromogen System, and cell nuclei were stained with hematoxylin. In addition, osteoid-producing HOS cells (NOS-1, NOS-2) and HMOS-P cells were analyzed in triplicate by FCM using CytoFLEX^TM^ (Beckman Coulter, Inc., Brea, CA, USA). The cells were incubated with 1:50 dilution of antibody against CAVIN1/PTRF for 20 min at 4 °C. The second antibody, Alexa 488 anti-rabbit IgG (1:1000 dilution), was incubated for 20 min at 4 °C.

Western blot (WB) analysis was performed as follows. Total proteins of HMOS-P and NOS-1 were extracted using a RIPA Lysis Buffer System (Santa Cruz Biotechnology). The proteins were mixed with loading SDS sample buffer (Thermo Fisher Scientific) containing 5% 2-mercaptoethanol, electrophoresed through 12.5% e-PAGEL (ATTO Corporation, Tokyo, Japan), and transferred onto Immobilon-P Transfer PVDF Membranes (Merck KGaA, Darmstadt, Germany). The membranes were blocked with 0.3% non-fat dry milk/TBS-0.1% Tween20 for 30 min and probed overnight with target antibodies. The following antibodies were used: anti-CAVIN1/PTRF and β-actin (1:1000, Proteintech). The membranes were incubated with horseradish peroxidase-conjugated secondary antibody (1:5000, Proteintech), and the protein bands were visualized using Clarity Western ECL Substrate (BIO-RAD). The Amersham™ ImageQuant™ 800 system (Cytiva, Tokyo, Japan) was used for chemiluminescence detection and gel imaging.

### 2.9. Statistical Analysis

Each experiment was performed in triplicate. Data are presented as the mean ± standard deviation (SD) and were analyzed using Welch’s *t*-test and Spearman’s rank correlation coefficient. Statistical Package for Social Science (SPSS) Statistics 24 (IBM, New York, NY, USA) was used to perform the statistical analyses. A *p*-value < 0.05 was considered to indicate statistical significance.

## 3. Results

### 3.1. Antibody Selection at Fluorescence Probe-Based ELISA

**Figure 1:**
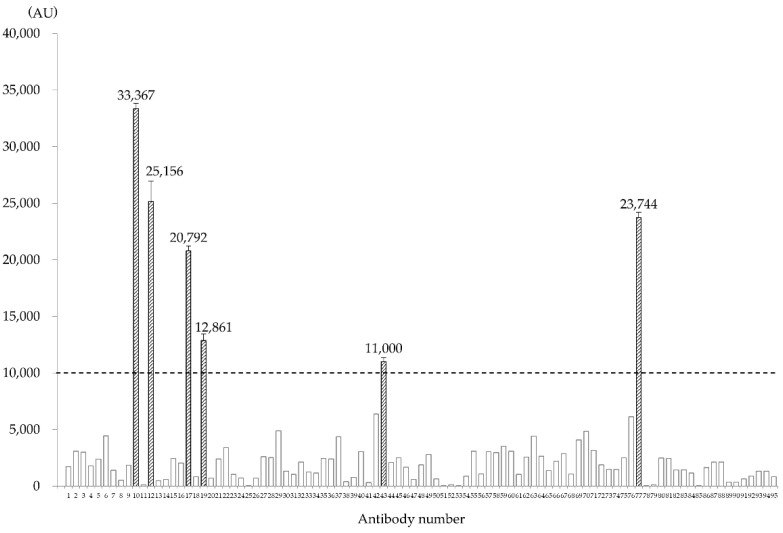
Measurement of fluorescence intensity of the 95 clones prepared by the fourth screening of the secondary human multicentric osteosarcoma (HMOS-P) using fluorescence probe-based enzyme-linked immunosorbent assay (ELISA). Among these, six clones showed a fluorescence intensity ≥ 10,000 arbitrary units (AU).

**Figure 2:**
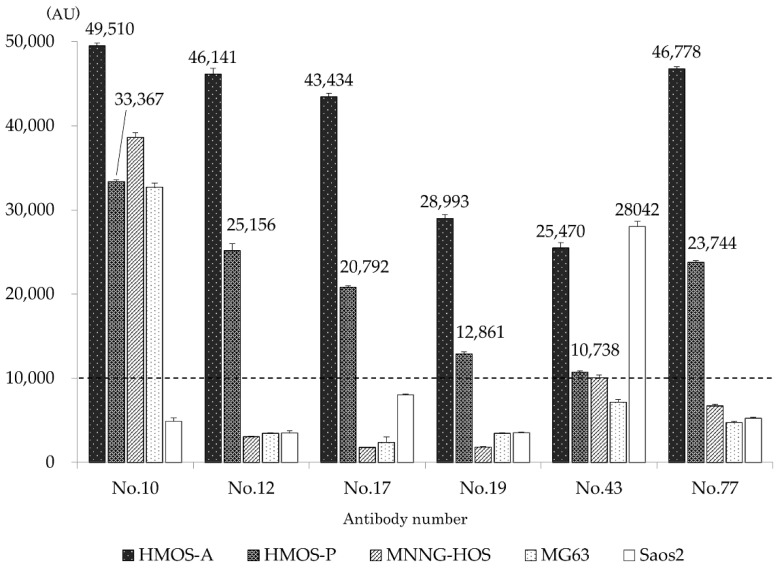
Comparison of antibody reactivities toward various cell lines. The six clones were also incubated with HMOS-P, the primary human multicentric osteosarcoma (HMOS-A), MNNG-HOS, MG-63, and Saos2, and the fluorescence intensity was measured using ELISA. All antibodies showed stronger fluorescence intensity, indicating greater reactivity with HMOS-A than with HMOS-P. Antibody numbers 10 and 43 were positive for MNNG-HOS. In MG63 cells, only antigen number 10 was positive, whereas only antigen number 43 was positive in Saos2 cells.

Among the generated antibody clones recognizing 95 independent antigens, antibody numbers 10, 12, 17, 19, 43, and 77 exhibited a fluorescence intensity ≥ 10,000 AU with HMOS-P and were therefore judged as positive (Figure 1). Antibody reactions using the ELISA method were performed on all cell types using the six antibodies (Figure 2). All antibodies exhibited stronger fluorescence intensity, indicating greater reactivity with HMOS-A than with HMOS-P. Antibody number 10 was positive for all cells except for Saos2 cells. Antibody numbers 12, 17, 19, and 77 were positive only for HMOS-A and HMOS-P, and the fluorescence intensity was less than 10,000 AU in the other HOS cell lines. Antibody number 43 was positive for HMOS-A, HMOS-P, and Saos2. The cell lines tested negative for mycoplasma infection.

### 3.2. Flow Cytometry

**Figure 3:**
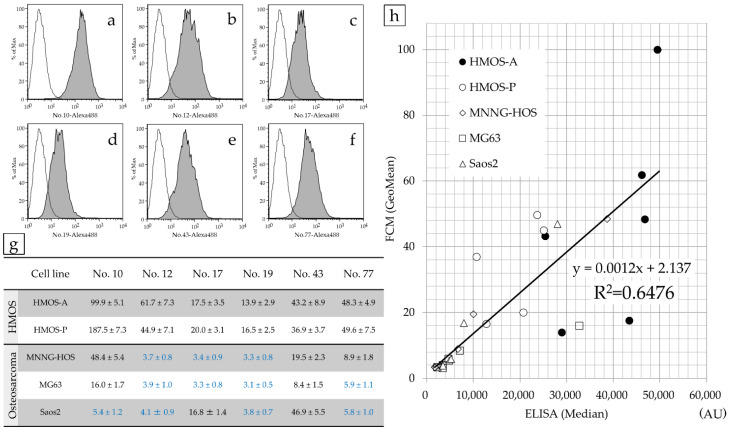
Correlation between ELISA-based and FCM fluorescence intensities at HMOS-P. FCM histograms of antibody numbers 10 (**a**), 12 (**b**), 17 (**c**), 19 (**d**), 43 (**e**), and 77 (**f**) incubated with HMOS-P. The FCM fluorescence intensity (GeoMean) for all cell lines was investigated. According to the results of flow cytometry of the HOS cell lines, most antibodies against antibody numbers 12, 17, 19, and 77 were negative (the values highlighted in blue were judged to be negative). In the HMOS cell line, most cells of antibody numbers 12, 43, and 77 were positive (**g**). The correlation between ELISA fluorescence intensity (median) and FCM fluorescence intensity (GeoMean) for all cell lines was strong. The correlation coefficient was r = 0.805 (**h**).

The positivity rate of FCM for HMOS-A, HMOS-P, MNNG-HOS, MG63, and Saos2 cells relative to their respective negative controls are listed in Table 1. The reactivity of each antibody with HMOS-P is depicted in FCM histograms (Figure 3a–f), and the GeoMean values for the fluorescence intensity of each cell type in FCM were compiled (Figure 3g).

The correlation between ELISA (median) and FCM fluorescence intensities (GeoMean) was investigated for all cell lines using Spearman’s rank correlation coefficient. We observed a significant correlation, with a correlation coefficient of 0.805 (*p* < 0.05).

### 3.3. Immunohistochemistry

**Figure 4:**
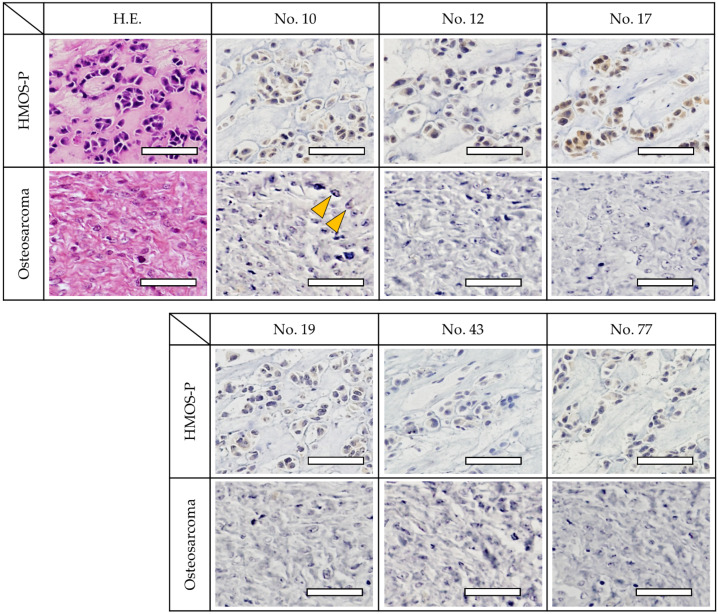
Immunohistochemistry of cancer tissues. Staining of similar paraffin sections of HMOS (upper row) and HOS (lower row). The cell lines exhibited strong positive staining with antibody numbers 10, 12, 17, and 77. The staining was also positive for antibody numbers 19 and 43 but relatively weaker. Immunohistochemical staining shows positive cells (yellow arrowheads). These results were comparable to those of the ELISA fluorescence intensity. Scale bar, 50 μm.

The immunostaining results using tissue samples from the HMOS posterior lesion site exhibited strong positive staining of the cells with antibody numbers 10, 12, 17, and 77. Antibody numbers 19 and 43 also revealed positive reactions, albeit with weaker staining. Using HOS tissue samples, a weak reaction was observed with antibody number 10; however, the other antibodies were negative (Figure 4).

### 3.4. Identification of Antigen Reacting with Antibody

**Figure 5:**
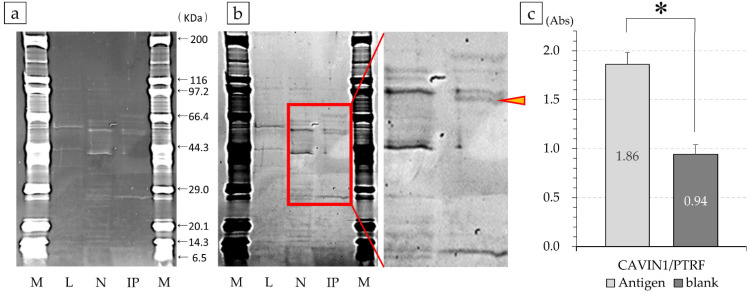
SDS-PAGE of the antigen–antibody complex and confirmation analysis with purified protein. Using immunoprecipitation through magnetic beads, the band incubated separately with the antibody was detected. Number 12 (**a**); M, marker; L, cell lysate solution; N, negative; IP, positive samples recovered by immunoprecipitation. Figure 5a is inverted in black and white and shows an enlarged view of more specific bands. This band (yellow arrowhead) was analyzed by Liquid chromatography-mass spectrometry (LC/MS) (**b**). The reactivity of commercially available CAVIN1/PTRF with antibody number 12 was confirmed. The protein had a stronger reactivity with the purified protein compared to that with the negative control, suggesting the occurrence of an antigen–antibody reaction (* *p* < 0.001) (**c**).

Based on the results of ELISA fluorescence intensity (median), FCM fluorescence intensity (GeoMean), and immunohistochemistry, we selected antibody number 12, which was highly reactive in HMOS and weakly reactive in all HOS. Subsequently, antibody number 12 was added to the cell lysates. In antibody numbers 12, a band of immune complexes, in which an antigen and a secondary antibody were bound to the antibody, was detected (Figure 5a,b). LC/MS analysis of this band revealed that antibody 12 corresponded to CAVIN1/PTRF, respectively. Bands detected in the IP that were different or stronger than those detected in the negative control (with only the antibody) were also analyzed, but no specific proteins were detected in the MASCOT data analysis.

Therefore, to confirm the reactivity of antibody number 12, its absorbance was compared against commercially available purified protein using ELISA. The absorbance of antibody number 12 against the CAVIN1/PTRF antigen was 1.860 ± 0.121, and the antigen was detected. The non-solidified negative was 0.943 ± 0.098, which was determined to be significantly different by Welch’s *t*-test (*p* < 0.001; Figure 5c).

**Figure 6:**
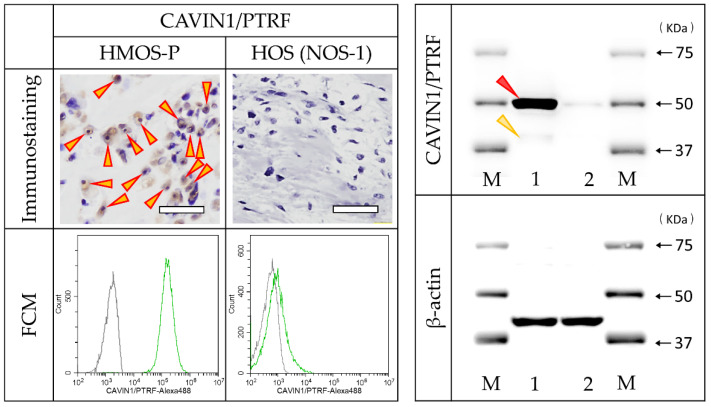
Immunostaining, FCM, and Western blot (WB) as a validation experiment for CAVIN1/PTRF. Tissue immunostaining of HMOS and HOS (upper row, red/yellow arrowheads indicate positive cells) and FCM of HMOS-P and NOS-1 cells (lower row) using the commercial antibody for CAVIN1/PTRF. The WB analysis result showed a specific and strong band (red arrowhead) of CAVIN1/PTRF. A very weak band at approximately 43 kDa, as calculated molecular weight, was also detected (yellow arrowhead). M, marker; 1, cell lysate of HMOS-P solution; 2, cell lysate of HOS solution. Scale bar = 50 μm.

Immunostaining with CAVIN1/PTRF antibody revealed positive cells in HMOS tissues but no positive cells in HOS tissue. FCM using CAVIN1/PTRF antibody revealed all-positive HMOS-P cells, slightly positive NOS-1 cells (6.8%), and all-negative NOS-2 cells. The WB analysis revealed a specific and strong band of CAVIN1/PTRF at 50 kDa in HMOS-P. On the other hand, a very weak band was observed in HOS at 50 kDa. The WB results were very similar to the FCM results (Figure 6).

## 4. Discussion

In this study, we comprehensively analyzed HMOS-P surface antigens using a phage display method and established a high-throughput antibody selection method using fluorescent ELISA, which is simpler than conventional FCM-based methods. We identified one protein (CAVIN1/PTRF) strongly expressed in the HMOS cell lines. In validation analysis using a specific commercial antibody that reacted with the identified protein, the antibody reacted specifically with HMOS tissue and not with the new HOS cell line. The cellular protein CAVIN1/PTRF, which was identified in this study, has not been described in HMOS or HOS. This protein is anticipated to develop into a novel molecular target marker for HMOS, for which there is neither a test nor an efficient treatment.

Human antibodies are expected to have clinical applications as anti-tumor drugs [24]. HMOS-A showed a higher positivity rate than HMOS-P cells; therefore, these cells were used to generate the antibody. This suggests that the antigens (proteins) recognized by the six antibodies were more strongly expressed in HMOS-A than in HMOS-P. This difference in the expression levels might be attributed to chemotherapy; notably, HMOS-A cells were obtained prior to chemotherapy, and HMOS-P cells were obtained from metastatic lesions that developed during chemotherapy treatment. Consequently, the expression levels in HMOS-P may have been slightly lower due to chemotherapy. However, the result of this selection is a shared protein across HMOS stages, as it was expressed in both HMOS-A and HMOS-P from primary and metastatic lesions, respectively. When FCM and ELISA were performed using the six antibody types, a strong correlation was observed between ELISA fluorescence intensity (median) and FCM fluorescence intensity (GeoMean) in HMOS-A and HMOS-P cells. This indicates that the ELISA-based fluorescence measurements from the developed method are likely valuable for screening phage antibodies. These results present a high-throughput method for selecting human antibodies that react with highly expressed proteins.

Antibody number 12 recognized CAVIN1/PTRF. CAVIN1/PTRF is a caveolar structural protein. Previously, CAVIN1 was termed Cav-P60 and reported [30,31] to form caveolae, an Ω structure on the cell membrane, with Caveolin-1. Caveolae is a cell membrane microdomain involved in regulating intracellular signaling pathways. Caveolae formation and maintenance were initially thought to be primarily due to the caveolin 21 kD protein. Three homologous genes are associated with caveolae expression in mammalian cells: *Cav1*, *Cav2*, and *Cav3*. The resultant proteins share a common topology. Caveolin is synthesized as a monomer and transported to the Golgi apparatus. Caveolins then combine with lipid rafts to form oligomers. These oligomerized caveolins form caveolae. The presence of caveolae results in localized changes in membrane morphology [32]. PTRF is a soluble protein containing a nuclear localization signal and a PEST domain imported into the cell nucleus by nuclear transport [33]. Moreover, PTRF (Cavin1) forms a trimer with Cavin2 and Cavin3 in caveolae formation and interacts with other membrane-bound proteins such as EHD2 and caveolae [34]. PTRF interacts with ZNF148 [35], and ZNF148 interacts with p53 [36]. Furthermore, the role of PTRF in cellular senescence reportedly depends on its interaction with caveolin-1, which appears to be regulated by targeting the caveolae and phosphorylation of PTRF, a regulator of cellular senescence acting through the p53/p21 and caveolae pathways [37]. Caveolin-1 reportedly functions as a tumor suppressor in sarcomas [38,39,40]. To the best of our knowledge, there have been no prior reports on the action of CAVIN1 in HOS.

The tissue specificity of CAVIN1/PTRF is relatively low, but it is expressed in mesenchymal cells, especially in adipose tissue, and has been reported as a prognostically unfavorable marker in urothelial cancer, ovarian cancer, and colorectal cancer. Since CAVIN1/PTRF is a central component of the CAVIN complex, and CAVIN1/PTRF is essential for caveolae complex formation [41], it interacts with a variety of proteins and genes. Caveolin-1 (Cav1) and CAVIN1/PTRF are components of caveolae, and Cav1 isoforms alpha and beta and BMP receptors BR1a and BR2 have been reported to co-localize at the cell surface [42]. Furthermore, the localization of BMP receptors in membrane domains has been associated with distinct endocytosis pathways that specifically affect Smad-dependent and Smad-independent signaling cascades [43].

In Ewing’s sarcoma, hypermethylation was identified in the body of the PTRF gene and in the CpG located in the S-Shore, correlating with transcriptional repression status. When the membrane caveolae of Ewing’s sarcoma cells are restored by the reintroduction of CAVIN1/PTRF, the MDM2/p53 complex is disrupted, resulting in p53 activation and induction of apoptosis [40].

In addition, the lysophosphatidic acid (LPA) receptor LPAR1 is highly expressed in common osteosarcoma cells. It was found that when osteosarcoma cells come into contact with platelets in the bloodstream, platelets are activated, and the invasive potential is enhanced via LPA produced and released from the activated platelets, resulting in lung metastasis. This indicates that LPAR1 antagonist administration may inhibit pulmonary metastasis of osteosarcoma [44]. CAVIN1/PTRF, which is specifically expressed in HMOS as revealed in this study, should also be investigated with antagonists.

The phage library method allowed us to rapidly narrow down the functional molecules in HMOS. The human antibody against CAVIN1/PTRF detected in this study may be clinically applicable as an anti-tumor drug. However, our study has some limitations. Our method is not suitable for the analysis of exclusively expressed intracytoplasmic proteins and we did not verify gene expression using RNA sequencing analysis. We were unable to perform confirmation outside of our own experience with HMOS cases, as HMOS is a very rare disease, and no cell lines were established from HMOS-derived cells. Although research on biomarkers has progressed remarkably, our method proves that it is possible to analyze not only genes but also expressed proteins, and furthermore, that the detected proteins can be recognized as antigens and antibodies can be produced. It has been very time-consuming and laborious to select and produce antibodies and to identify the antigens recognized by these antibodies. We believe that our newly developed method overcomes these weaknesses. In addition, we will continue to explore the possibilities for clinical application of antibody–drug conjugate for CAVIN1/PTRF, which were found in this study, in the treatment of HMOS or the prevention of bone metastasis. Because we investigated a rare case of HMOS, the antibody needs to be validated in a number of other HMOS cases for potential application in testing and treatment approaches.

## 5. Conclusions

We performed a comprehensive antibody screening of HMOS-P surface antigens using the phage display method and developed an antibody selection technique using fluorescence-based ELISA. The results of this ELISA technique exhibited a strong correlation with those of the conventional FCM method, which allowed the identification of a human antibody that reacts specifically with HMOS tissue samples. CAVIN1/PTRF was observed to specifically bind to HMOS cell lines. We believe that this protein may be used as potential biomarker in the diagnosis or treatment of HMOS.

## Figures and Tables

**Table 1:** Positivity rate of flow cytometry (FCM) for HMOS-A, HMOS-P, MNNG-HOS, MG63, and Saos2 cells.

Cell Line	No. 10	No. 12	No. 17	No. 19	No. 43	No. 77
HMOS-A	90.3 ± 1.1	88.9 ± 1.5	88.8 ± 1.5	76.1 ± 1.4	92.1 ± 1.0	92.9 ± 1.1
HMOS-P	77.5 ± 2.1	78.8 ± 2.3	77.1 ± 1.9	63.2 ± 2.2	77.8 ± 2.0	82.9 ± 1.7
MNNG-HOS	96.8 ± 0.9	3.4 ± 0.2	0.3 ± 0.1	0.2 ± 0.1	91.1 ± 1.4	45.4 ± 5.1
MG63	76.4 ± 1.5	1.0 ± 0.1	0.2 ± 0.1	0.2 ± 0.1	41.5 ± 6.3	24.7 ± 2.1
Saos2	22.3 ± 1.9	0.6 ± 0.2	68.2 ± 3.6	0.7 ± 0.2	96.1 ± 0.7	14.4 ± 1.7

## Data Availability

Not applicable.

## Supplementary Materials

The following supporting information can be downloaded at: https://www.mdpi.com/article/10.3390/cancers14235829/s1, Figure S1: Two novel HMOS cell lines; Table S1: Phage Library Screening for HMOS-P.
