# Supplementary material for: A Novel High-Throughput Screening Method for a Human Multicentric Osteosarcoma-Specific Antibody and Biomarker Using a Phage Display-Derived Monoclonal Antibody
Source: Cancers (Basel). 2022 Nov 26;14(23):5829. doi: 10.3390/cancers14235829 (PMC9739802; doi:10.3390/cancers14235829)
Supplement: Supplementary file 1 [file cancers-14-05829-s001.zip › cancers-1980828-supplementary.pdf]

cancers MDPI

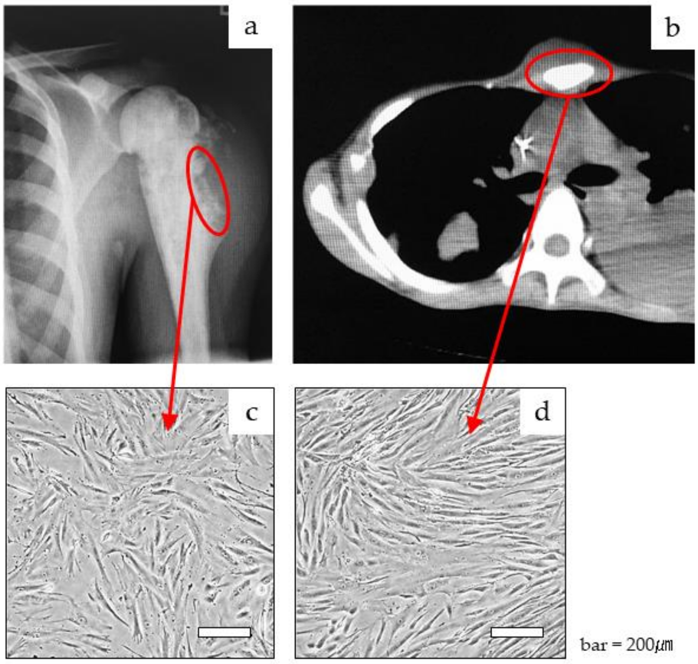

Figure S1. Two novel HMOS cell lines. Tumor tissues were harvested from the primary lesions of the upper arm (primary lesions) (**a**) and the newly emerging, late sternal lesions during treatment (**b**). Multicentric osteosarcoma cell lines were established at HMOS-A (**c**) and HMOS-P (**d**). Scale bar, 200 μm (reference 8).

Table S1. Phage Library Screening for HMOS-P. A comprehensive screening of antibodies against HMOS-P was performed using the AIMS7 phage library. Finally, four screenings were performed, concentration was performed until the denominator of the recall rate was 103, and 95 clones of antibodies were selected.

Round	Wet Weight (Cells)	Input	Output	Recovery
**First**	110 μL → 60 μL	3.79×10^{13}	4.25×10^{8}	$1/8.92 \times 10^{4}$
**Second**	115 μL → 70 μL	4.89×10^{10}	1.88×10^{5}	$1/2.60 \times 10^{5}$
**Third – 1**	65 μL → 35 μL	1.28×10^{9}	1.40×10^{4}	$1/9.14 \times 10^{4}$
**Third – 2**	110 μL → 65 μL	1.30×10^{9}	2.32×10^{4}	$1/5.60 \times 10^{4}$
**Fourth**	155 μL → 90 μL	1.35×10^{9}	3.07×10^{6}	$1/4.40 \times 10^{3}$
